# Perceived support, social and marital challenges in the lives of breast cancer survivors after illness: a self-administered cross-sectional survey

**DOI:** 10.3389/fsoc.2023.1227529

**Published:** 2023-09-07

**Authors:** Walaa Ammar-Shehada, Khaled Abusaman, Piet Bracke

**Affiliations:** ^1^Health and Demographic Research, Ghent University, Ghent, Belgium; ^2^Independent Researcher, Gaza, Palestine

**Keywords:** breast cancer, Gaza, Palestine, social support, health inequities

## Abstract

**Background:**

Breast cancer (BC) is a traumatic illness. BC is the leading female cancer in Palestine. Complex socio-political dynamics impact patients’ lives, resulting in an increasing need for social support to develop resilience after illness.

**Methods:**

Data was collected through a cross-sectional survey targeting women living in the Gaza Strip who had been diagnosed with BC. The survey was self-administered and distributed to 350 women between 1 March and 30 May 2021. Descriptive statistics and multinomial logistic regression analysis (SPSS, version 28.0) were used to explore perceived support, post-illness social and marital changes and the association between these changes and socio-demographic, illness-related and support-related variables.

**Findings:**

About four-fifth of the women with BC felt supported after illness, either fully or partially, mainly by family members, non-governmental organisations, spouses, and peers. Nevertheless, nearly half of the women perceived negative social changes after illness, and about 40% of married or formerly married women perceived negative changes in their marital life. Survivors’ lived experiences varied by age, marital status, motherhood, prescribed treatment (specifically mastectomy), and the absence of informal support in social life and lack of partner support amongst married or formerly married women.

**Conclusion:**

This study shows how BC undermines the social status of women and further exacerbates existing social vulnerabilities. Nevertheless, it is possible to manage and potentially overturn this circumstance by enveloping patients with social support. Guiding partners, families, and friends on providing emotional and instrumental support will help survivors to cope better during recovery.

## 1. Introduction

Breast cancer (BC) is a traumatic illness in the lives of women and can impact their functioning. It is the most diagnosed cancer worldwide and represents one in four cancers amongst women globally (International Agency for Research on Cancer, 2020). Although BC occurrence in high-income countries is twice as frequent as it is in low-and-middle-income countries (78.3 and 36.5 per 100,000, respectively), the proportion of death amongst patients is the same in both types of countries (12.9 and 12.8 per 100,000 respectively) (International Agency for Research on Cancer, 2018). Cancer and its treatment can cause considerable physical and emotional distress. Patients constantly face the fear of death and endure pain, stress, and financial burdens. Thus, social support after diagnosis is crucial for coping and maintaining overall quality of life (Adam and Koranteng, 2020). Having and maintaining a solid support network can help individuals navigate post-illness challenges and promote their physical, emotional, and social well-being (Dukes Holland and Holahan, 2003).

### 1.1. Social support and other attributes

Previous studies looked into the changes in the lives of BC survivors, especially in relation to perceived support. Such support varies from assistance through a social network, which is often informal or voluntary, to formal support provided by health-related or societal organisations and could include guidance, monetary or psychological support (Albrecht and Goldsmith, 2003). “The literature indicates that social support systems are particularly important protective factors for individuals experiencing a stressful event and that protective factors are necessary for the process of resilience to occur” (Zhang et al., 2017). It is a primary interpersonal resource that has been consistently found to be associated with psychological well-being in times of stress (Norris and Kaniasty, 1996). Social support is one of three processes of the relational content of microsocial relationships, along with social regulation and social conflict (House et al., 1988). Many stressful experiences can be linked to social structures and a person’s position within them. This is because social structures can shape the way people interact with each other, access resources, and experience diverse levels of power and privilege. These processes work through the social structure of a set of relationships with two elements: social integration and social network structure (Norris and Kaniasty, 1996).

It is evident in the literature that larger social networks and greater social support are related to a higher quality of life after a diagnosis of BC (Zhang et al., 2017). The extent of social support is associated with the quantity and quality of social networks that depend on belonging to specific social groups (e.g., BC peers) or enjoying specific social status (e.g., married). Hence people in a relationship have access to greater social support, which in turn promotes better well-being (Stronge et al., 2019). Social support, including positive emotional and information help provided by a partner, has been found to be an important detector of health-related quality of life amongst women with BC (Leung et al., 2014). A supportive partner can provide emotional and practical support and opportunities for social engagement and activities that can help foster a sense of connection and belonging. Moreover, being in a relationship can also provide stability and security, which can help reduce stress and anxiety.

As social support is a feature in the lives of survivors, previous studies have looked into the impact of other features, such as medical and socio-demographic factors. Many studies have shown that factors like age, marital status, education and financial situation have a significant association with the overall quality of life and influence its differential domains (Arora et al., 2001; Cui et al., 2004; Kayl and Meyers, 2006; Meneses et al., 2012; Konieczny et al., 2020).

### 1.2. Study context

#### 1.2.1. Health and cancer care

Constituting 18.6% of all cancer cases in Palestine, BC is the leading female cancer there. In the Gaza Strip (GS), its incidence rate is 34.5 per 100,000 females (MoH, 2022). The five-year survival of BC patients in the GS between 2005 and 2014 was 65.1% and it is in line with estimates from surrounding Arab countries, but it was lower than in developed Mediterranean countries (e.g., in Italy or in Jewish people in Israel) (Panato et al., 2018). BC represents 30% of cancer deaths amongst women in reproductive age in 2021 and cancer ranked as the second cause of death after COVID-19 as the first cause of death amongst this group of women in the same year (MoH, 2022). The reason behind this steep rise is structural. The political instability, including the 16-year blockade (started in 2007), impacted the development and functionality of health institutions and the broader social determinants of health. First, there are no organised screening programmes, and the detection of BC is determined by personal and social factors (United Nations Population Fund-UNFPA, 2020); hence significant part of BC patients are diagnosed at an advanced stage (Panato et al., 2018). Second, the provision of health services is fragmented amongst multiple healthcare providers, so the treatment trajectory of cancer patients could include various places within and/or outside the GS. This makes the treatment pathway without definite time bound due to barriers to access diagnostic and treatment services. Third, there is no central administration of medical files, so patients have to seek assistance and services from multiple providers on the basis of their access to information, knowledge, awareness and social networks, with no guided follow-up. Fourth, COVID-19 has intensified long-standing, politically driven vulnerabilities amongst Palestinians in the last three years, which was mainly tangible in the reduction of services across the health sector, which has delayed treatment and resulted in the reduction of referrals out of GS (Alser et al., 2020). That has negatively impacted women’s mental health and general well-being (Medical Aid for Palestinians-MAP-UK, 2020). Fifth, Illness in Gaza imposes additional challenges on patients compared to peers in neighbouring countries due to structural barriers to accessing health services. According to the latest Right to Health report released by the World Health Organization in oPt, 55% was the average availability of essential medicines in Gaza in 2019, which together with lack of sub-specialties services, continue to keep cancer care as the most common reason for referrals from 2019 to 2021 (26% of the total referral) [World Health Organization (WHO), 2023]. Referrals mean referring patients outside the Palestinian public hospitals to receive needed care. That is in practice a lengthy referral and access procedure in the cases of referral to a destination hospital outside the GS. The complexity of the procedure and decisions to be taken by multiple actors do not guarantee to reach the destination hospital on time. In 2021, only 62% of health referral permits were approved [World Health Organization (WHO), 2023], adding additional challenges to patients’ treatment trajectories.

#### 1.2.2. Socio-economic context:

Social structure in the GS is complex and can operate in different directions based on the individual’s social position. The traditional social structure is primarily based on tribal and family ties. The family unit is highly valued and is often the primary source of support for individuals. The family system is generally organised around the extended family, which typically consists of several generations, including parents and grandparents, living together or in proximity. In most cases, it is a patrilocal system unless the husband (or couple) has the financial means (or work-related arrangements) to establish a family independently in a different place. It is common for family members to pool their resources to help each other. Since it is a patriarchal society, men enjoy higher authority and, the male head of the family is typically the decision-maker and authority figure. However, many women also play an essential role in family life and decision-making. They are often responsible for domestic work and childcare and contribute to the family income in various ways. Women’s authority varies based on multiple factors; age is an example, so older women are generally in a more powerful position than younger women. Other factors, such as marital or economic status, intersect to change the power dynamics affecting and surrounding women. The support mechanisms include informal networks of friends, as well as charitable organisations, community-based organisations, and religious institutions. However, the ongoing instability, poverty – more than half of Gaza’s population lives under the poverty line (53%) (World Bank Group, 2020), and unemployment rates that reached 47% [Palestinian Central Bureau of Statistics (PCBS), 2022] have significantly impacted the social context, causing widespread trauma and social dislocation. This has led to the emergence of new forms of support, such as Non-Governmental Organisations (NGOs) and international aid agencies, which resulted in a complex interplay of traditional and modern social structures and a mix of formal and informal support mechanisms.

In these contextual settings, women remain less privileged than men. Although more females had educational attainment of a secondary degree and above than males (47.8 and 41.4%, respectively) in 2020 [Palestinian Central Bureau of Statistics (PCBS), 2021a], yet women continue to be economically excluded, despite their high education levels. Education is not enough to increase the rate of women’s participation in the labour force, and it is also not enough to ensure job security for women seeking employment (United Nations – ESCWA, 2023). In the context of low female labour force participation, women’s economic dependence on their husbands or other male family members is a risk factor for psychological, physical, and sexual violence (United Nations – ESCWA, 2023). Financial vulnerability and other social norms promote early marriage, so women become financially dependent on their partners. The median age at first marriage in Palestine was 20.7 years for females and 25.3 years for males in 2019 [Palestinian Central Bureau of Statistics (PCBS), 2021b]. Although the percentage of early marriage (under 18) decreased compared to previous years, 19.3% of marriages in Gaza in the year 2020 involved girls under 18 (United Nations – ESCWA, 2023) which contributes to the social vulnerability of women.

Illness adds to the vulnerability of individuals, especially in impoverished socio-economic conditions where they have to bargain and chase health services, not because oPt is one of the low-and-middle-income, but because of the beforehand explained context of chronic emergency for decades. The Palestinian community is characterised by high solidarity and informal support dynamics (Kharroubi and Abu Seir, 2016). This support is intensified in major life events. Nevertheless, social pressure can be an important issue in such a small geographical area (362 square kilometres) with a high population density (more than two million people). For instance, BC is often viewed as related to femininity and can threaten marriage or affect chances of marriageability. Alaloul et al. found that 95.4% of the women surveyed were anxious that their husbands would divorce them if they underwent a mastectomy (Alaloul et al., 2019), however, the study could not validate the reality of such fears.

*The main purpose* of this paper is to explore the sources and types of support received by BC survivors, in addition to understanding whether survivors are experiencing changes in their social and marital life due to perceived support as well as differences in socio-demographic factors, illness and treatment-related factors. It also aims to explore how the survivors perceive the change (as positive or negative) if it does exist compared with their life before the illness.

## 2. Methods

Data were collected through a self-administered cross-sectional survey that targeted women with BC living in the GS. The survey was distributed between 1 March and 30 May 2021 to 350 women. The estimated number of BC patients in the GS (the research population) was 2,058 women. With a confidence level of 95%, 324 is the recommended sample size. The initial draft of the questionnaire was developed by the first author in consultations with co-authors. The questionnaire was validated. The construct, content and face validity of the survey was checked by three public health experts, and the comments received were considered in the survey prior to piloting it with ten women. The survey language was the local language in Palestine, Arabic. The first page included an introduction to the survey, its purpose and the main eligibility criteria, followed by a consent statement. If a woman agreed to complete the survey and checked the consent box, she was forwarded to the next section of the survey. The link of the final survey was shared with the administration of NGOs for dissemination amongst BC survivors. The target group of this study is cancer survivors, according to the definition of The American Cancer Society, which refers to anyone who has ever been diagnosed with cancer, no matter where they are in the course of their disease (American Cancer Society, n.d.). The inclusion criteria were: (1) females diagnosed with BC at least once, (2) and living in the GS during illness and treatment. The 350 women were all reachable through the lists compiled by NGOs. Four NGOs cooperated in recruiting cancer survivors registered in their database. The NGOs were selected based on geographical distribution to include survivors from all governorates. The survey was electronic and was shared through closed social media groups (mailing lists, WhatsApp and SMS).

This paper will analyse data on support and changes in survivors’ lives. The survey collected data in five sections; socio-demographic, illness history, treatment history and place, types and sources of support received during treatment and changes in survivors’ lives after illness. The response rate was 71%, with 251 responses (out of *N* = 350), one was excluded, and 250 were valid and included in the analysis.

### 2.1. Dependent variables

*Change in social life* was assumed to be positive, negative, or non-existent.

Positive social change after illness was observed in a higher level of socialisation and interactions with others and being able (and having the opportunity) to speak out about their illness. In addition, the patients had the opportunity to receive physical and psychological support and be surrounded by love, respect, and protection.

Negative social change after illness reflected stigmatisation and feelings of pity from others as well as the tendency of ill women to feel lonely and internalise feelings. The patients felt insecurity caused by illness, for example, by viewing it as a threat to their source of income.

The survey participants were given the definition (and examples) of positive and negative changes and then asked to answer with “Yes,” “No” or “No Change.”

Change in the marital relationship was assumed to be either positive, negative, or non-existent.

Positive change in the marital relationship after illness was manifested through increased emotional support and solidarity in moments of weakness as well as more help from the spouse in household work and childcare; the sexual relationship was not negatively impacted, and the spouse showed respect for the changes that the wife was undergoing.

A negative change in the marital relationship after illness took the form of less sex or an end to the sexual relationship, negative expressions or voicing of dissatisfaction due to physical changes in the wife’s body or complaints related to her tiredness. It could include abandoning her in the marital bed or in a separate room, or the wife might be sent to her family’s house for them to take care of her during the treatment period; It could also be seen in a spouse’s failure to show respect and offer support with home chores.

After the positive and negative change to women had been defined, the survey participants were asked to answer with “Yes,” “No” or “No Change.”

### 2.2. Independent variables

Independent variables were grouped into three main sections: socio-demographic variables, illness and treatment history, and support-related variables.

#### 2.2.1. Socio-demographic information

Socio-demographic information such as governorate, age, marital status, number of children, employment, education, refugee status and place of residence.

Governorates: The GS comprises five governorates: North, Gaza, Middle, Khan Yunus and Rafah.

Age distribution: The survey was open to women with the criteria mentioned earlier; however, the respondents were between 26 and 70 years. Age is the second biggest BC risk factor after gender. The incidence rate of BC increases significantly with age and reaches its peak at the age of menopause. None of the survey respondents was below 26 years old, which was expected as BC is rare in women younger than 25 years. The participants were divided into four groups: 26–40, 41–50, 51–65 and > 65, taking into account the periods of high fertility and menopause.

Marital status: Married, single, widow, divorced and separated were the options available to respondents. Later, during the analysis, separated and divorced were merged into one group. The divorced women had formalised everything through the court and were legally divorced, while separated ones were still married on paper but, in reality, were no longer married.

Number of Children: The grouping of 0, 1–4 and > 5 children was based on the average size of households in the GS, which was 5.7 individuals (including parents) in 2018 [Palestinian Central Bureau of Statistics (PCBS), 2019].

Employment: Employed women and non-employed.

Education: The education system in the GS consists of primary, preparatory and secondary levels, followed by a higher university degree. Primary education is until the 6th grade, preparatory until the 9th grade, and secondary until the 12th grade.

Palestinian Refugees: These are officially defined by UNRWA as “persons whose normal place of residence was Palestine during the period from 1 June 1946 to 15 May 1948, and who lost both home and means of livelihood because of the 1948 conflict” (UNRWA, 2022). In the context of the GS and West Bank (WB), Palestinian refugees are more internally displaced people and do not fit into the global mainstream definition of refugees who flee their country. However, given the specificity of the clause, UNRWA was established and mandated by the UN General Assembly to serve” Palestine refugees” in five operational areas: Jordan, Lebanon, Syria, the GS, and WB, including East Jerusalem (UNRWA, 2022).

Place of residence: city or camp.

#### 2.2.2. History of illness and treatment

This included the number of times BC had been diagnosed, stage at diagnosis, prescribed treatment, breast reconstruction surgery, health status at the time of completing the survey, place of treatment, types of health facilities accessed and financial coverage. Frequency of BC diagnosis: how often the BC had been diagnosed. The stage at diagnosis: The survey participants were asked to identify the stage (I, II, III, IV) that the BC had attained when diagnosed. During the piloting phase of the survey, some women did not know this, so “Did not know” was added as a possible answer in the final survey. Prescribed treatment: Women chose which treatment protocols were proposed by health providers: surgery, chemotherapy, radiotherapy, hormonal, or a combination. Surgery where all possible options of lumpectomy, radical bilateral or unilateral mastectomy, and partial bilateral or unilateral mastectomy were listed. Breast Reconstruction surgery: This is surgery to rebuild the shape and look of the breast after women undergo surgery as part of their BC treatment (American Cancer Society, 2022). Place of treatment: Were the women treated in the GS, outside the GS, or both in and out of the GS? Health status at the time of completing the survey: The choice was from: Was the respondent still under treatment, had she recovered, or had she finished the treatment but was still making follow-up visits, or had not started the treatment yet.

#### 2.2.3. Support-related variables

There were two aspects under this heading: types and sources. The types of support covered any help or aid that an ill woman received, such as assistance to access needed information and financial, social or psychological support. The women were considered to have been given instrumental support if they received information and/or financial help or both. They were considered to have received emotional support if they were given psychological and/or social–emotional support.

Sources of support: The women were considered to have received formal support when it came from governmental institutions, NGOs, or both. They were considered to have received informal support when it came from friends and neighbours, BC peers or a family member. Partner support was labelled an independent variable separate from other family members in the analysis of marital change, given that it is an evident factor affecting the marital relationship.

### 2.3. Data analysis

Statistical Package for the Social Sciences (SPSS) (version 28.0) was used for statistical analysis. Descriptive frequency analysis was used to present an overview of the socio-demographic characteristics, illness and treatment history, types and sources of support received and changes in survivors’ lives. This was followed by using logistic models to evaluate the association between the first dependent variable (change in social life) and the independent socio-demographic, illness-related and treatment-related and support-related variables for all respondents (*N* = 250). Multinomial logistic regression was used to determine the relationship between the second dependent variable (change in marital life) and the independent socio-demographic, illness and treatment-related and support-related variables for married or formerly married women (*N* = 213).

### 2.4. Ethical approval

The Helsinki Committee for Ethical Approval of the Palestinian Health Research Council provided written approval for carrying out this research (approval PHRC/HC/730/20). An electronic survey was used for data collection because of the movement restrictions and physical distancing measures imposed during the COVID-19 pandemic, and online consent statement was essential to start completing the survey. The administrations of four NGOs working with BC patients approved and supported the distribution of survey forms through their lists and social media groups. The questionnaire was validated before sharing with the administration of NGOs for distribution. The three authors of this study had access to the collected data. Confidentiality and anonymity were maintained while collecting, storing and analysing data.

## 3. Results

### 3.1. Participants’ characteristics

Participants’ characteristics of the sample are reported in Tables 1, 2. As can be seen, slightly less than three-quarters of respondents are married (73%, 182/250), and 12% (29/250) are either separated or divorced. Most participants are mothers (76%, 189/250). The sample’s median of total illness years was 5 years, and treatment years was 4 years. Surgery was the highest prescribed treatment, where 96% (239/250) had undergone surgery as part of their prescribed treatment protocols. Low utilisation of breast construction surgery was prominent, whereas only one woman had undergone the procedure.

**Table 1:** Number and percentage of socio-demographic, illness-related, and treatment-related variables (*N* = 250).

Variable	Number	%	Variable	Number	%
Socio-demographic variables			Illness-related variables		
*Governorate*			*Number of detection times*		
North	28	11.2	Once	180	72
Middle	51	20.4	Twice	55	22
Khan Yunus	38	15.2	More than two times	15	6
Rafah	46	18.4			
Gaza	87	34.8	*Place of treatment*		
			in the GS*	74	29.6
*Age*			in and outside the GS	164	65.6
26–40	68	27.2	outside the GS	12	4.8
41–50	61	24.4			
51–65	106	42.4	*Stage-at-diagnosis*		
> 65	15	6.0	Stage I	73	29.2
			Stage II	82	32.8
*Marital status*			Stage III	39	15.6
Widow	19	7.6	Stage IV	7	2.8
Single	20	8.0	Did not know	49	19.6
Married	182	72.8			
Separated/divorced	29	11.6	*Treatment protocols*		
			Radiotherapy	162	64.8
*Educational level*			Chemotherapy	213	85.2
Primary/preparatory	25	10	Hormonal	169	67.6
Secondary	156	62.4	Surgical	239**	95.6
Undergraduate/postgraduate	69	27.6			
			*Breast reconstruction surgery*		
*Number of children*			No	245	98.0
0	41	16.4	Yes	1	0.4
1–4	83	33.2	Did not know	4	1.6
5–11	106	42.4			
NA (not previously married)	20	8	*Health status when completing the survey*		
			Under treatment	166	66.4
Employment			Recovered	10	4
*Employed*	28	11.2	Finished treatment & undergo check-ups	73	29.2
Not employed	222	88.8	Other	1	0.4
*Refugee/non-Refugee*			* 77% in public hospitals, 23% in NGOs, 48% in private hospitals and clinics. ** (12%) 29/239 Lumpectomy, (71%) 170/239 Radical bilateral or unilateral mastectomy, (17%) 40/239 Partial bilateral or unilateral mastectomy.		
Refugee	172	68.8			
Non-refugee	78	31.2			
		
*Residential area*					
Camp	85	34			
City	165	66			

**Table 2:** Number of years with illness and treatment (*N* = 250).

Number of years with illness		Median = 5
	Number	Percentage
<=5 years	144	58%
6 years – 10 years	78	31%
>10	28	11%
*Total*	*250*	*100%*
Total number of treatment years		Median = 4
<=5 years	162	65%
6 years – 10 years	69	28%
>10	19	8%
*Total*	*250*	*100%*

Table 3 summarises the numbers and percentages of the types and sources of support as per the survey responses. Most of the respondents perceived receiving diverse types of support (80%, 200/250), either fully (47%, 117/250) or partially (33%, 83/250). Emotional support was perceived to be received at 70% (176/250) to be higher than instrumental support (64%, 161/250). The family was regarded as the primary source of support for 63% (157/250), followed by NGOs at 52% (131/250) and husbands at 49% (104/213).

**Table 3:** Types and sources of support received (*N* = 250).

Variable	Number	%
Support received		
Yes	117	46.8
Sometimes	83	33.2
No	50	20
Types of support		
All	41	16.4
Instrumental (*Financial and/or information*)	161	64.4
Emotional *(Psychological and/or social)*	176	70.4
Sources of support		
Public sector	6	2.4
NGOs	131	52.4
Peer support	92	36.8
Friends/Neighbours	65	26.0
Family	157	62.8
Husband*	104*	48.8*

*N = 213: applicable only for current or formerly married women.

Table 4 presents the perceived changes after illness in both social and marital life. Almost half of the participants perceived negative change in their social life (47%, 118/250), while 30% (75/250) experienced no change. When it comes to perceived changes in marital life, 40% (85/213) perceived negative change and 43% (91/213) perceived no change after the illness.

**Table 4:** The perceived change in survivors’ social and marital lives.

Change	Social life (*N* = 250)		Marital life (*N* = 213)	
	Number	Percent	Number	Percent
Non-existent	75	30%	91	43%
Positive	57	23%	37	17%
Negative	118	47%	85	40%
*Total*	*250*	*100*	*213*	*100*

### 3.2. Multinomial logistic regression analysis

Table 5 presents the results of a multinomial logistic regression analysis that examined the positive and negative changes in the social life of women with BC using “women who reported no change” as a reference category. This was the final round of analysis that included the set of variables that were significant in perceived changes. The initial model of analysis that included all independent variables is presented in Appendix 1.

**Table 5:** Multinomial logistic regression analysis of changes in survivors’ social life by socio-demographic and illness-related variables (*N* = 250).

	Change in social life^a^		Positive				Negative			
			Beta (β)	Standard error	*p* value	Odds ratio	Beta (β)	Standard error	*p* value	Odds ratio
*Socio-demographic variables*										
	Number of children		0.25	0.09	0.00	1.28	0.13	0.08	0.09	1.14
	Age	> 65	0.04	0.80	0.96	1.04	−2.66	1.18	0.02	0.07
		26–40	0.66	0.57	0.2	1.94	1.58	0.52	0.00	4.86
		41–50	0.31	0.53	0.56	1.37	0.61	0.48	0.20	1.84
		51–65	0^b^				0^b^			
	Marital status	Widow	−0.80	0.94	0.40	0.45	0.170	0.71	0.81	1.19
		Single	1.62	0.80	0.04	5.07	−0.436	0.84	0.60	0.65
		Separated/Divorced	1.21	0.92	0.19	3.34	2.186	0.82	0.01	8.90
		Married	0^b^				0^b^			
	Educational level	Primary/Preparatory	0.58	0.79	0.47	1.78	0.749	0.74	0.31	2.16
		Secondary	0.35	0.48	0.47	1.42	0.302	0.43	0.49	1.35
		Undergraduate or postgraduate	0^b^				0^b^			
*Illness-related and treatment-related variables*										
	Number of detection times	Once	−0.704	0.845	0.405	0.5	0.198	0.830	0.81	1.22
		Twice	−0.454	0.936	0.628	0.64	0.766	0.900	0.40	2.15
		More than two times	0^b^				0^b^			
Prescribed treatment	Hormonal	No	−0.277	0.437	0.526	0.79	−0.568	0.405	0.16	0.57
		Yes	0^b^				0^b^			
	Radiotherapy	No	0.166	0.520	0.749	1.18	0.273	0.469	0.56	1.31
		Yes	0^b^				0^b^			
	Chemotherapy	No	0.150	0.615	0.807	1.16	−0.945	0.634	0.14	0.39
		Yes	0^b^				0^b^			
	Surgical treatment	No	−0.911	1.207	0.450	0.40	−0.298	1.045	0.776	0.742
		Yes	0^b^				0^b^			
	Underwent mastectomy	No	−1.686	1.013	0.096	0.185	−1.973	0.852	0.021	0.139
		Yes	0^b^				0^b^			
	Type of surgical procedure	Partial mastectomy	−0.849	1.090	0.436	0.428	−3.058	0.934	0.001	0.047
		Radical mastectomy	−1.004	1.046	0.337	0.366	−1.839	0.857	0.032	0.159
		Lumpectomy	0^b^				0^b^			
	Place of treatment	Outside the GS	1.116	0.837	0.182	3.052	−1.384	1.009	0.170	0.251
		In the GS	0.560	0.528	0.289	1.751	−0.774	0.503	0.124	0.461
		In and outside the GS	0^b^				0^b^			
*Support-related variables*										
Source of support	Formal Support	No	−0.763	0.434	0.078	0.466	−0.678	0.401	0.090	0.508
		Yes	0^b^				0^b^			
	Informal support	No	−1.115	0.917	0.224	0.328	1.477	0.664	0.026	4.379
		Yes	0^b^				0^b^			

a The reference category is: ‘No Change.’

b This parameter is set to zero because it is redundant.

The final model shows that when the number of children of married/or previously married women increased by one, the chance of experiencing positive change increased by 1.28 compared with women who reported no change (OR = 1.28, *p* = 0.00). Women aged 26 to 40 years old were more likely to experience negative change (OR = 4.86, *p* = 0.002), while the opposite was found amongst women aged above 65 years (OR = 0.07, *p* = 0.02). Furthermore, single women were more likely to experience positive change compared with married women (OR = 5.07, *p* = 0.04), unlike separated/divorced women, who were likely to experience negative change (OR = 8.90, *p* = 0.01). It is noticeable that women who did not undergo mastectomy (OR = 0.14, *p* = 0.02) were less likely to experience negative social change compared to women who had a mastectomy. Finally, women who reported an absence of informal support were experiencing more negative change (OR = 4.38, *p* = 0.03).

Table 6 summarises the results of multinomial logistic regression analysis to identify the positive and negative changes in the marital life of BC survivors, with “women who reported no change” as a reference category. This is considered the final model after running an initial model presented in Appendix 2 that included all independent socio-demographic, illness-related, treatment-related and support-related variables. The final model included only the set of variables that appeared to be significant regarding changes in social and marital life in the initial analysis.

**Table 6:** Multinomial logistic regression analysis of changes in survivors’ marital life by socio-demographic, illness-related and support-related variables (*N* = 213*).

	Change in marital relationship^a^		Positive				Negative			
			Beta (β)	Standard error	*P* value	Odds ratio	Beta (β)	Standard error	*P* value	Odds ratio
	*Socio-demographic variables*									
	Number of children		0.270	0.113	0.017	1.310	−0.015	0.081	0.852	0.985
	Age	> 65	2.004	1.220	0.101	7.418	0.029	1.088	0.978	1.030
		26–40	2.227	0.731	0.002	9.275	1.725	0.610	0.005	5.612
		41–50	2.061	0.661	0.002	7.850	1.532	0.530	0.004	4.626
		51–65	0^b^				0^b^			
	Marital status	Widow	−0.712	1.438	0.621	0.491	−2.403	1.253	0.055	0.090
		Separated/divorced	−0.247	1.455	0.865	0.781	1.514	0.896	0.091	4.544
		Married	0^b^				0^b^			
	Educational level	Primary and preparatory	1.947	1.017	0.056	7.006	−1.100	0.913	0.228	0.333
		Secondary education	1.576	0.686	0.022	4.834	0.723	0.495	0.144	2.060
		Undergraduate or postgraduate	0^b^				0^b^			
	*Illness and treatment-related variables*									
	Number of detection times	Once	−1.095	0.944	0.246	0.334	−1.228	0.940	0.191	0.293
		Twice	−1.882	1.072	0.079	0.152	−0.179	0.983	0.855	0.836
		More than two times	0^b^				0^b^			
Prescribed treatment	Hormonal	No	1.699	1.457	0.244	5.467	−0.258	1.380	0.851	0.772
		Yes	0^b^				0^b^			
	Radiotherapy	No	−0.709	0.627	0.258	0.492	0.154	0.436	0.724	1.166
		Yes	0^b^				0^b^			
	Chemotherapy	No	−1.123	0.731	0.125	0.325	−0.833	0.556	0.134	0.435
		Yes	0^b^				0^b^			
	Surgical treatment	No	2.080	0.880	0.018	8.005	2.226	0.776	0.004	9.261
		Yes	0^b^				0^b^			
	Underwent mastectomy	No	−0.490	1.249	0.695	0.612	−3.104	1.184	0.009	0.045
		Yes	0^b^				0^b^			
	Type of surgical procedure	Partial mastectomy	0.539	1.332	0.686	1.714	−1.284	0.947	0.175	0.277
		Radical mastectomy	1.810	1.327	0.172	6.113	−0.467	0.871	0.592	0.627
		Lumpectomy	0^b^				0^b^			
	Place of treatment	Outside the GS	1.064	0.983	0.279	2.898	−1.680	1.411	0.234	0.186
		In the GS	0.192	0.649	0.767	1.212	−1.160	0.593	0.050	0.314
		In and outside the GS	0^b^				0^b^			
	*Support-related variables*									
Source of support	Formal Support	No	0.554	0.528	0.294	1.740	−0.073	0.436	0.867	0.930
		Yes	0^b^				0^b^			
	Informal support	No	0.075	0.600	0.900	1.078	0.643	0.476	0.177	1.902
		Yes	0^b^				0^b^			
	Partner support	No	−0.393	0.610	0.519	0.675	1.688	0.460	0.000	5.406
		Yes	0^b^				0^b^			

a The reference category is: ‘No Change.’

b This parameter is set to zero because it is redundant.

* *N* = 213. The table summarises the analysis of 213 respondents out of 250. Thirty-seven women had the answer to this question about the change of marital relationship as inapplicable since they had not been married when diagnosed with BC.

The model shows that when the number of children of married or formerly married women increased by one, the chance of experiencing positive change increased by 1.31, compared with women who reported no change (OR = 1.31, p = 0.02). It is noticeable that women who had not had mastectomy were less likely to experience negative marital change (OR = 0.05, p = 0.01) compared with those who had undergone a mastectomy. Finally, when it comes to support-related variables and association with the change in marital relationships, the absence of partner support was significant in more negative marital relationships (OR = 5.41, *p* = 0.00).

## 4. Discussion

This paper has explored the types and sources of support perceived by BC survivors and examined how diverse socio-demographic characteristics, illness-related and treatment-related factors, and perceived support can affect survivors’ social life and marriage, and if the change exists, what direction it takes. Although the present study shows that about four-fifth of women with BC perceived one or more than one type of support after illness, either fully or partially, yet BC had a worse impact on women who are already in vulnerable positions according to the local culture and social structure. The lived experiences of women after illness and perceived change varied according to the socio-demographic characteristics, mainly age, marital status, motherhood and prescribed treatment, specifically surgery, and by sources of support, particularly partner support.

### 4.1. Women as kin-keepers and the consequences of BC

Motherhood is a privilege for married women in the Palestinian community. Having children is a prominent source of strength and hope for mothers with BC and gives a sense of purpose and motivation. “Women are the glue holding social relations together. They play a central role as friends, daughters, sisters, mothers, and grandmothers throughout all stages of the life course” (Bracke et al., 2008). This role applies to mothers in the Palestinian context, where they are assigned the primary childcare role. Thus, they view fighting illness and resilience as essential to carrying out their motherhood mission. Looking after kin is primarily a female activity that is frequently passed from mother to daughter (Rosenthal, 1985). It is proved in the literature that younger women tend to have a greater responsibility than older women with BC for maintaining the well-being of others in their family and for providing stability in everyday family life (Coyne and Borbasi, 2007). In the Palestinian community, mothers with adult sons/daughters are cared for during illness; and the adult children also help do house chores that are often seen as the mother’s primary duty. So, mothers whose family members have grown up bear less burden than mothers with young children. This aligns with Sammarco’s study, which highlights the higher need for support to the partners and children of young BC survivors to enhance their quality of life (Sammarco, 2001). In both cases, if a young mother with BC sees her motherhood as a source of resilience or if she receives support from adult children, it affects her life positively during the treatment trajectory. This all could explain the results of the mother survivor’s perception of positive change after the illness. This is in line with previous research by Wilson that examined the inter-relationship between illness and key sources of identity (mainly motherhood) and showed that the respondents’ emphasis on their need to survive and protect their children represented a fundamental reformulation of their identities as mothers (Wilson, 2007).

### 4.2. Single, divorced, and married women and the consequences of BC within the patriarchal kinship system

The situation of single women in Palestine is complex and varied. As with all individuals, the experiences of single women in Gaza depend on many factors, including age, social status, employment and family background. Same applied to divorced/separated women with additional challenges: financial insecurity, social isolation, and legal and logistical challenges of obtaining a divorce. They may also face stigma and discrimination, particularly if they do not have support from their families or are divorced in a community where divorce is rarely accepted, and marriage failure is attributed to women. Despite these challenges, some strong and resilient women work to build better lives for themselves. As for BC survivors, and as presented in the results section, single women tend to experience more positive change after illness due to the belief that their lives will not be impacted as much as those of married women because they do not have to worry about a husband’s perception of and reaction to BC. In other words, they perceive BC as an illness directly connected to women’s femininity (Helms et al., 2008), and that is not an area of worry for unmarried survivors because femininity is mainly connected to the marriage institution and the role of the wife.

Divorced/separated women appeared in the analysis to experience a more negative change in social life, partly because some had been separated because of their illness (Community Media Centre, n.d.). In Palestine, the spouse is the main actor, especially in critical decisions like divorce. Women seldom have the choice of an exit strategy from marriage. Men are in a powerful position in the local gendered kinship systems and can end the marriage or find an alternative when the wife is ill (for instance, through a second marriage), particularly because of the support of legal and religious institutions to male partners. Often, it becomes the primary responsibility of the wife’s family to take care of their ill daughter. So, while women cope with their physical and mental disturbance due to illness, they also have to deal with the social pressure resulting from their separation, abandonment or divorce, which in turn changes their lives to the worse, unless they have other sources of power through employment or support received by the family.

### 4.3. Mastectomy and self-image

The less invasive the treatment procedure, the better the social outcome. The less negative effect was significant amongst respondents who did not undergo mastectomy compared to those who underwent it. There could be several reasons for such negative experiences. Women undergoing mastectomy may face a negative change socially because they may experience a loss of physical appearance and body image (Koçan and Gürsoy, 2016). The loss of a breast can be a significant and visible change, affecting how a woman feels about her appearance and identity, that they are no longer competent, especially with the low utilisation of breast reconstruction surgery (only one respondent underwent it out of 250). Some women who undergo mastectomy may feel self-conscious or anxious about their appearance and feel that they are no longer attractive or feminine (Arroyo and López, 2011). They may experience a loss of self-esteem and confidence (Sukartini and Permatasari, 2020). Moreover, the surgery and recovery process can be physically and emotionally demanding, and some women may feel that they are no longer able to do things that they used to do due to physical complications. Finally, women undergoing mastectomy may face negative change because of the stigma and misunderstanding surrounding BC and mastectomy (Tripathi et al., 2017). Some people might not understand why a woman had chosen to undergo a mastectomy, or they may have misconceptions about BC and its treatment. This could lead to social isolation and discrimination, which could be difficult for a woman already dealing with BC’s physical and emotional challenges. All these reasons can be contributing factors to the experience of post-illness negative change amongst survivors who underwent a mastectomy.

### 4.4. (Un)supportive partners

Support is a well-known determinant of well-being. Previous studies (Pistrang and Barker, 1995; Borstelmann et al., 2015; Salakari et al., 2017) have reinforced the spouse’s central role during the BC recovery phase. Although a high proportion of respondents stated that their family was the main source of support, followed by NGOs, then by husband, but when the partner’s support was absent, it was a significant element in the negative change of marital relationship. This aligns with previous research concerning marital dynamics where the partner was proved to be key in maintaining and supporting intimacy, which includes closeness, sharing, and sexual functioning, or damaging it (Holmberg et al., 2001). So, the results show that devastating consequences could result in the life of married survivors to non-supportive partners than single survivors. Moreover, in the context of this study, the scarcity of governmental support is also a crucial factor. Only 2.4% of women perceived support from the government; hence, the absence of overarching governmental ‘social security’ system when marriage is impacted negatively exacerbates the fears and psychological distress amongst BC survivors. The results clearly depict the utmost importance of partner support for the relationship quality of women having BC. Thus, it seems the lack of support from the partner is especially detrimental, and the content of the support of less relevance.

## 5. Conclusion and recommendations

This paper shows how BC undermines the position of women in society because of the local gendered kinship systems. The study contributes to the field by theoretically and empirically recognising the heterogeneity of BC survivors and how they experience uncertainties and changes in their social and marital life according to their socio-demographic factors and social support. It concludes that BC patients are not a hegemonic group; thus, approaching them collectively should take into account individual social differences. Therefore, when designing interventions, the study encourages policymakers to consider social factors at the diagnosis level and include the promotion of self-value and self-love amongst women who are less supported according to the embedded social roles. Moreover, targeted care and more resources should be invested in offering instrumental and social–emotional support based on individual needs assessment to ensure that the needs of patients and their families are met, with more attention to women undergoing surgical procedures to ensure the maintenance of the post-illness quality of life. Additionally, this study shows the importance of raising awareness about BC facts and symptoms amongst patients and their social networks, including families and partners. Approaching patients’ families and guiding them on how to continue offering the support needed by patients will strengthen survivors’ resilience by making them feel loved and protected, which is vital for maintaining the quality of life after illness. At the research level, this study recommends more in-depth qualitative studies to unpack the social dynamics surrounding BC patients in a complex and interrelated community like Gaza. This would guide future interventions aiming at strengthening protection mechanisms and minimising potential social injustice that could arise after an illness through strengthening survivors’ resilience and improving the well-being of BC patients during the treatment trajectory.

## 6. Limitations

The survey was self-administered, and due to the COVID-19 situation in the GS and the imposed restrictions, it had to be in electronic form. That means some technologically illiterate women would depend on another person, such as a daughter, son, or spouse, to fill in the form. That would limit the respondent’s privacy, especially in answering questions related to social life and marital relationship changes.

The respondents are women registered in NGOs lists, which means that, in one way or another, they reached out to NGOs and could have joined support groups and attended awareness sessions. Such groups are more open to discussion and aware of sources of support. Those participants are somehow more vocal and empowered than women who have not interacted with NGOs. Women who were not participating in NGOs’ activities were left behind and were not sufficiently represented in the sample. Additionally, the fact that this study is targeting BC survivors, as only alive, capable and reachable women with BC can fill in the survey, might have led to a distortion of the sample in such a way that there were fewer advanced-stage respondents.

## Data availability statement

The datasets generated and analyzed during this study are not publicly available. Reasonable requests for access can be made to the corresponding author who will consider any such requests. Requests to access the datasets should be directed to Walaa.Shehada@Ugent.be.

## Ethics statement

The study involving human participants was approved by The Committee for Ethical Approval of the Palestinian Health Research Council provided (approval number: PHRC/HC/730/20). Informed consent was obtained from the participants and confirmed digitally.

## Author contributions

WA-S was responsible for study design, conceptualisation, data collection, validation, analysis, and writing and reviewing the manuscript. KA supported the data check and validation, advised on the statistical analysis, results’ conceptualisation, and presentation. PB critically reviewed the manuscript, advised on the conceptualisation, methodology, and presentation of the results, and provided overall supervision. All authors gave final approval for the manuscript to be published. All authors have seen and approved the final version of the abstract and manuscript for publication.

## Conflict of interest

The authors declare that the research was conducted in the absence of any commercial or financial relationships that could be construed as a potential conflict of interest.

## Publisher’s note

All claims expressed in this article are solely those of the authors and do not necessarily represent those of their affiliated organizations, or those of the publisher, the editors and the reviewers. Any product that may be evaluated in this article, or claim that may be made by its manufacturer, is not guaranteed or endorsed by the publisher.
