# Supplementary material for: Perceived support, social and marital challenges in the lives of breast cancer survivors after illness: a self-administered cross-sectional survey
Source: Front Sociol. 2023 Sep 7;8:1227529. doi: 10.3389/fsoc.2023.1227529 (PMC10513897; doi:10.3389/fsoc.2023.1227529)
Supplement: Supplementary file 1 [file Table_1.docx]

Supplementary Material

Perceived Support, Social and Marital Challenges in the Lives of Breast Cancer Survivors after Illness: A Self-Administered Cross-Sectional Survey

Walaa Ammar-Shehada^*^, Khaled Abusaman, Piet Bracke

*** Correspondence:** Corresponding Author: [Walaa.Shehada@UGent.be](mailto:Walaa.Shehada@UGent.be)

# Supplementary Tables

## APPENDIX 1

| **Table 1: Multinomial logistic regression analysis of survivors’ changes in social life by socio-demographic, illness-related and support-related variables (N=250)** | | | | | | | | | | | | | | | | | | | |
| --- | --- | --- | --- | --- | --- | --- | --- | --- | --- | --- | --- | --- | --- | --- | --- | --- | --- | --- | --- |
| **Change in social life** | | | **Positive** | | | | | | | | **Negative** | | | | | | | | |
|  |  |  | Beta (β) | Standard Error | Wald | df | *P* value | Odds Ratio (OR) | 95% Confidence Interval for OR | | Beta (β) | Standard Error | Wald | df | *P* value | Odds Ratio (OR) | 95% Confidence Interval for OR | | |
| **Socio-demographic variables** | | | | | | | | | | | | | | | | | | |  |
| Number of children | | | 0,268 | 0,096 | 7,843 | 1 | 0,005 | 1,308 | 1,084 | 1,578 | 0,163 | 0,084 | 3,766 | 1 | 0,052 | 1,177 | 0,998 | 1,388 | |
| Age | > 65 | | 0,125 | 0,875 | 0,020 | 1 | 0,886 | 1,133 | 0,204 | 6,296 | -2,872 | 1,215 | 5,585 | 1 | 0,018 | 0,057 | 0,005 | 0,613 | |
|  | 26-40 | | 0,679 | 0,653 | 1,080 | 1 | 0,299 | 1,972 | 0,548 | 7,095 | 1,764 | 0,596 | 8,752 | 1 | 0,003 | 5,835 | 1,814 | 18,776 | |
|  | 41-50 | | -0,134 | 0,592 | 0,051 | 1 | 0,821 | 0,875 | 0,274 | 2,790 | 0,441 | 0,520 | 0,719 | 1 | 0,397 | 1,554 | 0,561 | 4,304 | |
|  | 51-65 | | 0^b^ |  |  | 0 |  |  |  |  | 0^b^ |  |  | 0 |  |  |  |  | |
| Marital status | Widow | | -0,646 | 1,059 | 0,372 | 1 | 0,542 | 0,524 | 0,066 | 4,175 | 0,375 | 0,778 | 0,233 | 1 | 0,629 | 1,456 | 0,317 | 6,691 | |
|  | single | | 2,024 | 0,881 | 5,278 | 1 | 0,022 | 7,571 | 1,346 | 42,576 | -0,004 | 0,883 | 0,000 | 1 | 0,996 | 0,996 | 0,176 | 5,623 | |
|  | divorced/separated | | 1,426 | 1,000 | 2,032 | 1 | 0,154 | 4,160 | 0,586 | 29,541 | 2,572 | 0,888 | 8,386 | 1 | 0,004 | 13,094 | 2,296 | 74,663 | |
|  | married | | 0^b^ |  |  | 0 |  |  |  |  | 0^b^ |  |  | 0 |  |  |  |  | |
| Employment | Employed | | 1,656 | 0,815 | 4,130 | 1 | 0,042 | 5,237 | 1,061 | 25,857 | 0,993 | 0,734 | 1,829 | 1 | 0,176 | 2,699 | 0,640 | 11,375 | |
|  | Not employed | | 0^b^ |  |  | 0 |  |  |  |  | 0^b^ |  |  | 0 |  |  |  |  | |
| Educational level | prim/prep | | 0,978 | 0,887 | 1,215 | 1 | 0,270 | 2,658 | 0,467 | 15,115 | 1,286 | 0,812 | 2,509 | 1 | 0,113 | 3,617 | 0,737 | 17,756 | |
|  | secondary | | 1,040 | 0,581 | 3,197 | 1 | 0,074 | 2,828 | 0,905 | 8,840 | 0,738 | 0,514 | 2,063 | 1 | 0,151 | 2,091 | 0,764 | 5,725 | |
|  | university and higher | | 0^b^ |  |  | 0 |  |  |  |  | 0^b^ |  |  | 0 |  |  |  |  | |
| Refugeehood status | Refugee | | 0,124 | 0,576 | 0,046 | 1 | 0,829 | 1,132 | 0,366 | 3,502 | 0,047 | 0,492 | 0,009 | 1 | 0,923 | 1,048 | 0,400 | 2,750 | |
|  | Non-Refugee | | 0^b^ |  |  | 0 |  |  |  |  | 0^b^ |  |  | 0 |  |  |  |  | |
| Residential area | Camp | | 0,618 | 0,485 | 1,619 | 1 | 0,203 | 1,854 | 0,716 | 4,801 | 0,477 | 0,430 | 1,231 | 1 | 0,267 | 1,612 | 0,694 | 3,747 | |
|  | City | | 0^b^ |  |  | 0 |  |  |  |  | 0^b^ |  |  | 0 |  |  |  |  | |
| **illness and treatment-related variables** | | | | | | | | | | | | | | | | | | |  |
| Number of years with illness | | | -0,179 | 0,104 | 2,966 | 1 | 0,085 | 0,836 | 0,682 | 1,025 | -0,137 | 0,092 | 2,197 | 1 | 0,138 | 0,872 | 0,727 | 1,045 | |
| Number of treatment years | | | 0,146 | 0,109 | 1,768 | 1 | 0,184 | 1,157 | 0,933 | 1,434 | 0,109 | 0,099 | 1,210 | 1 | 0,271 | 1,115 | 0,919 | 1,353 | |
| Frequency of illness | | once | -0,909 | 0,942 | 0,932 | 1 | 0,334 | 0,403 | 0,064 | 2,551 | 0,371 | 0,886 | 0,175 | 1 | 0,676 | 1,449 | 0,255 | 8,233 | |
|  |  | twice | -0,361 | 1,052 | 0,118 | 1 | 0,731 | 0,697 | 0,089 | 5,477 | 1,259 | 0,989 | 1,619 | 1 | 0,203 | 3,520 | 0,507 | 24,467 | |
|  |  | more than twice | 0^b^ |  |  | 0 |  |  |  |  | 0^b^ |  |  | 0 |  |  |  |  | |
| Stage at diagnosis | | Stage I or II | 0,230 | 0,618 | 0,139 | 1 | 0,709 | 1,259 | 0,375 | 4,223 | -0,050 | 0,543 | 0,009 | 1 | 0,926 | 0,951 | 0,328 | 2,759 | |
|  | | Stage III or IV | 0,229 | 0,786 | 0,085 | 1 | 0,771 | 1,257 | 0,269 | 5,871 | 0,130 | 0,675 | 0,037 | 1 | 0,847 | 1,139 | 0,303 | 4,276 | |
|  | | Did not know | 0^b^ |  |  | 0 |  |  |  |  | 0^b^ |  |  | 0 |  |  |  |  | |
| Hormonal | | No | -0,329 | 0,461 | 0,509 | 1 | 0,476 | 0,720 | 0,292 | 1,776 | -0,630 | 0,427 | 2,179 | 1 | 0,140 | 0,532 | 0,231 | 1,230 | |
|  | | Yes | 0^b^ |  |  | 0 |  |  |  |  | 0^b^ |  |  | 0 |  |  |  |  | |
| Radiotherapy | | No | -0,178 | 0,560 | 0,101 | 1 | 0,751 | 0,837 | 0,280 | 2,507 | 0,027 | 0,493 | 0,003 | 1 | 0,957 | 1,027 | 0,391 | 2,699 | |
|  | | Yes | 0^b^ |  |  | 0 |  |  |  |  | 0^b^ |  |  | 0 |  |  |  |  | |
| Chemotherapy | | No | 0,026 | 0,669 | 0,001 | 1 | 0,969 | 1,026 | 0,276 | 3,810 | -1,053 | 0,691 | 2,325 | 1 | 0,127 | 0,349 | 0,090 | 1,351 | |
|  | | Yes | 0^b^ |  |  | 0 |  |  |  |  | 0^b^ |  |  | 0 |  |  |  |  | |
| Surgery | | No | -1,700 | 1,294 | 1,726 | 1 | 0,189 | 0,183 | 0,014 | 2,307 | -0,620 | 1,118 | 0,307 | 1 | 0,579 | 0,538 | 0,060 | 4,813 | |
|  | | Yes | 0^b^ |  |  | 0 |  |  |  |  | 0^b^ |  |  | 0 |  |  |  |  | |
| Mastectomy | | No | -1,346 | 1,008 | 1,782 | 1 | 0,182 | 0,260 | 0,036 | 1,877 | -2,005 | 0,884 | 5,148 | 1 | 0,023 | 0,135 | 0,024 | 0,761 | |
|  | | Yes | 0^b^ |  |  | 0 |  |  |  |  | 0^b^ |  |  | 0 |  |  |  |  | |
| Type of surgical procedure | | Partial bilateral or unilateral | -1,147 | 1,095 | 1,099 | 1 | 0,295 | 0,317 | 0,037 | 2,713 | -3,455 | 0,971 | 12,671 | 1 | 0,000 | 0,032 | 0,005 | 0,212 | |
|  | | Radical bilateral or unilateral | -1,154 | 1,047 | 1,215 | 1 | 0,270 | 0,315 | 0,040 | 2,455 | -2,021 | 0,884 | 5,228 | 1 | 0,022 | 0,133 | 0,023 | 0,749 | |
|  | | Lumpectomy | 0^b^ |  |  | 0 |  |  |  |  | 0^b^ |  |  | 0 |  |  |  |  | |
| Place of treatment | | Outside the GS | 1,307 | 0,890 | 2,157 | 1 | 0,142 | 3,695 | 0,646 | 21,147 | -1,468 | 1,070 | 1,881 | 1 | 0,170 | 0,230 | 0,028 | 1,877 | |
|  |  | In the GS | 0,815 | 0,563 | 2,098 | 1 | 0,147 | 2,259 | 0,750 | 6,806 | -0,638 | 0,525 | 1,476 | 1 | 0,224 | 0,528 | 0,189 | 1,479 | |
|  |  | In and outside the GS | 0^b^ |  |  | 0 |  |  |  |  | 0^b^ |  |  | 0 |  |  |  |  | |
| **Support-related variables** | | | | | | | | | | | | | | | | | | |  |
| Receiving support | No | | -0,374 | 0,598 | 0,390 | 1 | 0,532 | 0,688 | 0,213 | 2,223 | -0,069 | 0,494 | 0,019 | 1 | 0,890 | 0,934 | 0,354 | 2,459 | |
|  | Yes | | 0^b^ |  |  | 0 |  |  |  |  | 0^b^ |  |  | 0 |  |  |  |  | |
| Instrumental support | No | | 0,072 | 0,534 | 0,018 | 1 | 0,893 | 1,074 | 0,377 | 3,063 | 0,959 | 0,502 | 3,649 | 1 | 0,056 | 2,609 | 0,975 | 6,978 | |
|  | Yes | | 0^b^ |  |  | 0 |  |  |  |  | 0^b^ |  |  | 0 |  |  |  |  | |
| Emotional support | No | | -0,906 | 0,617 | 2,157 | 1 | 0,142 | 0,404 | 0,121 | 1,354 | 0,365 | 0,550 | 0,442 | 1 | 0,506 | 1,441 | 0,491 | 4,232 | |
|  | Yes | | 0^b^ |  |  | 0 |  |  |  |  | 0^b^ |  |  | 0 |  |  |  |  | |
| Formal support | No | | -0,597 | 0,505 | 1,400 | 1 | 0,237 | 0,550 | 0,205 | 1,480 | -0,983 | 0,473 | 4,328 | 1 | 0,037 | 0,374 | 0,148 | 0,945 | |
|  | Yes | | 0^b^ |  |  | 0 |  |  |  |  | 0^b^ |  |  | 0 |  |  |  |  | |
| Informal support | No | | -1,215 | 0,980 | 1,538 | 1 | 0,215 | 0,297 | 0,043 | 2,025 | 1,281 | 0,728 | 3,093 | 1 | 0,079 | 3,601 | 0,864 | 15,015 | |
|  | Yes | | 0^b^ |  |  | 0 |  |  |  |  | 0^b^ |  |  | 0 |  |  |  |  | |
| ^a^ The reference category is: ‘No Change’.  ^b^ This parameter is set to zero because it is redundant | | | | | | | | | | | | | | | | | | | |

**Supplementary Table 1.** Multinomial logistic regression analysis of survivors’ changes in social life by socio-demographic, illness-related and support-related variables (N=250)

## APPENDIX 2

| **Table 2: Multinomial logistic regression analysis of survivors’ changes in marital life by socio-demographic, illness-related and support-related variables (N=213*)** | | | | | | | | | | | | | | | | | |
| --- | --- | --- | --- | --- | --- | --- | --- | --- | --- | --- | --- | --- | --- | --- | --- | --- | --- |
| **Change in marital relationship** | | **Positive change in marital relationship** | | | | | | | | **Negative change in marital relationship** | | | | | | | |
|  |  | Beta (β) | Standard Error | Wald | df | *P* value | Odds Ratio (OR) | 95% Confidence Interval for OR | | Beta (β) | Standard Error | Wald | df | *P* value | Odds Ratio (OR) | 95% Confidence Interval for OR | |
| **Socio-demographic variables** | | | | | | | | | | | | | | | | | |
| Number of children | | 0,281 | 0,124 | 5,106 | 1 | 0,024 | 1,324 | 1,038 | 1,689 | -0,021 | 0,090 | 0,054 | 1 | 0,817 | 0,979 | 0,821 | 1,169 |
| Age | > 65 | 2,169 | 1,503 | 2,083 | 1 | 0,149 | 8,748 | 0,460 | 166,353 | 0,157 | 1,327 | 0,014 | 1 | 0,906 | 1,170 | 0,087 | 15,756 |
|  | 26-40 | 2,406 | 0,908 | 7,024 | 1 | 0,008 | 11,087 | 1,871 | 65,692 | 2,155 | 0,743 | 8,407 | 1 | 0,004 | 8,629 | 2,010 | 37,041 |
|  | 41-50 | 1,864 | 0,799 | 5,450 | 1 | 0,020 | 6,452 | 1,349 | 30,868 | 1,890 | 0,642 | 8,666 | 1 | 0,003 | 6,619 | 1,881 | 23,294 |
|  | 51-65 | 0^b^ |  |  | 0 |  |  |  |  | 0^b^ |  |  | 0 |  |  |  |  |
| Marital status | Widow | -0,093 | 1,849 | 0,003 | 1 | 0,960 | 0,911 | 0,024 | 34,142 | -2,596 | 1,359 | 3,651 | 1 | 0,056 | 0,075 | 0,005 | 1,069 |
|  | Separated/Divorced | -0,370 | 1,765 | 0,044 | 1 | 0,834 | 0,691 | 0,022 | 21,979 | 1,287 | 1,006 | 1,634 | 1 | 0,201 | 3,621 | 0,504 | 26,032 |
|  | Married | 0^b^ |  |  | 0 |  |  |  |  | 0^b^ |  |  | 0 |  |  |  |  |
| Employment | Employed | -1,126 | 1,405 | 0,642 | 1 | 0,423 | 0,324 | 0,021 | 5,092 | 0,925 | 0,777 | 1,415 | 1 | 0,234 | 2,521 | 0,550 | 11,560 |
|  | Not employed | 0^b^ |  |  | 0 |  |  |  |  | 0^b^ |  |  | 0 |  |  |  |  |
| Educational level | Primary/Preparatory | 2,529 | 1,248 | 4,108 | 1 | 0,043 | 12,538 | 1,087 | 144,631 | -0,900 | 1,043 | 0,746 | 1 | 0,388 | 0,406 | 0,053 | 3,138 |
|  | Secondary | 2,214 | 0,866 | 6,534 | 1 | 0,011 | 9,152 | 1,676 | 49,972 | 1,146 | 0,627 | 3,339 | 1 | 0,068 | 3,144 | 0,920 | 10,743 |
|  | Undergraduate or postgraduate | 0^b^ |  |  | 0 |  |  |  |  | 0^b^ |  |  | 0 |  |  |  |  |
| Refugeehood status | Refugee | -0,266 | 0,805 | 0,109 | 1 | 0,741 | 0,767 | 0,158 | 3,712 | 0,303 | 0,634 | 0,228 | 1 | 0,633 | 1,354 | 0,391 | 4,687 |
|  | Non-Refugee | 0^b^ |  |  | 0 |  |  |  |  | 0^b^ |  |  | 0 |  |  |  |  |
| Residential area | Camp | 0,303 | 0,693 | 0,191 | 1 | 0,662 | 1,354 | 0,348 | 5,261 | -0,008 | 0,492 | 0,000 | 1 | 0,987 | 0,992 | 0,378 | 2,602 |
|  | City | 0^b^ |  |  | 0 |  |  |  |  | 0^b^ |  |  | 0 |  |  |  |  |
| **illness and treatment-related variables** | | | | | | | | | | | | | | | | | |
| Total number of years with illness | | -0,092 | 0,162 | 0,318 | 1 | 0,573 | 0,912 | 0,664 | 1,255 | 0,066 | 0,097 | 0,457 | 1 | 0,499 | 1,068 | 0,883 | 1,292 |
| Total number of treatment years | | -0,048 | 0,177 | 0,074 | 1 | 0,785 | 0,953 | 0,674 | 1,347 | -0,179 | 0,112 | 2,564 | 1 | 0,109 | 0,836 | 0,671 | 1,041 |
| Frequency of illness times | once | -1,577 | 1,146 | 1,894 | 1 | 0,169 | 0,207 | 0,022 | 1,953 | -1,931 | 1,083 | 3,178 | 1 | 0,075 | 0,145 | 0,017 | 1,212 |
|  | twice | -1,913 | 1,274 | 2,256 | 1 | 0,133 | 0,148 | 0,012 | 1,792 | -0,445 | 1,139 | 0,153 | 1 | 0,696 | 0,641 | 0,069 | 5,967 |
|  | more than twice | 0^b^ |  |  | 0 |  |  |  |  | 0^b^ |  |  | 0 |  |  |  |  |
| Stage at diagnosis | Stage I or II | -0,680 | 0,908 | 0,561 | 1 | 0,454 | 0,506 | 0,085 | 3,005 | -0,978 | 0,666 | 2,158 | 1 | 0,142 | 0,376 | 0,102 | 1,387 |
|  | Stage III or IV | -2,109 | 1,211 | 3,035 | 1 | 0,082 | 0,121 | 0,011 | 1,302 | -0,652 | 0,807 | 0,653 | 1 | 0,419 | 0,521 | 0,107 | 2,533 |
|  | Did not know | 0^b^ |  |  | 0 |  |  |  |  | 0^b^ |  |  | 0 |  |  |  |  |
| Hormonal | No | -0,768 | 0,704 | 1,190 | 1 | 0,275 | 0,464 | 0,117 | 1,843 | 0,024 | 0,488 | 0,002 | 1 | 0,961 | 1,024 | 0,394 | 2,666 |
|  | Yes | 0^b^ |  |  | 0 |  |  |  |  | 0^b^ |  |  | 0 |  |  |  |  |
| Radiotherapy | No | -1,550 | 0,858 | 3,262 | 1 | 0,071 | 0,212 | 0,039 | 1,141 | -1,098 | 0,616 | 3,179 | 1 | 0,075 | 0,333 | 0,100 | 1,115 |
|  | Yes | 0^b^ |  |  | 0 |  |  |  |  | 0^b^ |  |  | 0 |  |  |  |  |
| Chemotherapy | No | 2,283 | 1,065 | 4,593 | 1 | 0,032 | 9,808 | 1,215 | 79,151 | 2,200 | 0,828 | 7,056 | 1 | 0,008 | 9,025 | 1,780 | 45,758 |
|  | Yes | 0^b^ |  |  | 0 |  |  |  |  | 0^b^ |  |  | 0 |  |  |  |  |
| Surgery | No | 1,332 | 1,699 | 0,615 | 1 | 0,433 | 3,790 | 0,136 | 105,893 | -0,636 | 1,418 | 0,201 | 1 | 0,654 | 0,529 | 0,033 | 8,520 |
|  | Yes | 0^b^ |  |  | 0 |  |  |  |  | 0^b^ |  |  | 0 |  |  |  |  |
| Mastectomy | No | 0,114 | 1,337 | 0,007 | 1 | 0,932 | 1,120 | 0,082 | 15,395 | -3,068 | 1,261 | 5,915 | 1 | 0,015 | 0,047 | 0,004 | 0,551 |
|  | Yes | 0^b^ |  |  | 0 |  |  |  |  | 0^b^ |  |  | 0 |  |  |  |  |
| Type of surgical procedure | Partial bilateral or unilateral | 0,820 | 1,455 | 0,318 | 1 | 0,573 | 2,270 | 0,131 | 39,296 | -1,076 | 1,048 | 1,055 | 1 | 0,304 | 0,341 | 0,044 | 2,657 |
|  | Radical bilateral or unilateral | 2,767 | 1,516 | 3,331 | 1 | 0,068 | 15,919 | 0,815 | 310,855 | -0,178 | 0,959 | 0,034 | 1 | 0,853 | 0,837 | 0,128 | 5,487 |
|  | Lumpectomy | 0^b^ |  |  | 0 |  |  |  |  | 0^b^ |  |  | 0 |  |  |  |  |
| Place of treatment | Outside the GS | 1,358 | 1,075 | 1,597 | 1 | 0,206 | 3,888 | 0,473 | 31,942 | -1,488 | 1,446 | 1,059 | 1 | 0,303 | 0,226 | 0,013 | 3,840 |
|  | In the GS | 0,109 | 0,733 | 0,022 | 1 | 0,882 | 1,115 | 0,265 | 4,693 | -1,364 | 0,668 | 4,161 | 1 | 0,041 | 0,256 | 0,069 | 0,948 |
|  | In and outside the GS | 0^b^ |  |  | 0 |  |  |  |  | 0^b^ |  |  | 0 |  |  |  |  |
| **Support-related variables** | | | | | | | | | | | | | | | | | |
| Receiving support | No | -0,438 | 0,743 | 0,348 | 1 | 0,555 | 0,645 | 0,150 | 2,768 | 0,483 | 0,574 | 0,707 | 1 | 0,400 | 1,621 | 0,526 | 4,999 |
|  | Yes | 0^b^ |  |  | 0 |  |  |  |  | 0^b^ |  |  | 0 |  |  |  |  |
| Instrumental support | No | 1,531 | 0,725 | 4,466 | 1 | 0,035 | 4,624 | 1,118 | 19,133 | 0,468 | 0,557 | 0,704 | 1 | 0,402 | 1,596 | 0,535 | 4,758 |
|  | Yes | 0^b^ |  |  | 0 |  |  |  |  | 0^b^ |  |  | 0 |  |  |  |  |
| Emotional support | No | -0,887 | 0,879 | 1,017 | 1 | 0,313 | 0,412 | 0,074 | 2,309 | -0,826 | 0,668 | 1,530 | 1 | 0,216 | 0,438 | 0,118 | 1,621 |
|  | Yes | 0^b^ |  |  | 0 |  |  |  |  | 0^b^ |  |  | 0 |  |  |  |  |
| Formal support | No | 1,039 | 0,646 | 2,587 | 1 | 0,108 | 2,827 | 0,797 | 10,029 | -0,038 | 0,516 | 0,006 | 1 | 0,941 | 0,962 | 0,350 | 2,647 |
|  | Yes | 0^b^ |  |  | 0 |  |  |  |  | 0^b^ |  |  | 0 |  |  |  |  |
| Informal support | No | -0,105 | 0,663 | 0,025 | 1 | 0,874 | 0,900 | 0,246 | 3,299 | 0,757 | 0,526 | 2,069 | 1 | 0,150 | 2,131 | 0,760 | 5,975 |
|  | Yes | 0^b^ |  |  | 0 |  |  |  |  | 0^b^ |  |  | 0 |  |  |  |  |
| Partner support | No | -0,964 | 0,823 | 1,370 | 1 | 0,242 | 0,381 | 0,076 | 1,916 | 2,009 | 0,580 | 11,977 | 1 | 0,001 | 7,454 | 2,390 | 23,251 |
|  | Yes | 0^b^ |  |  | 0 |  |  |  |  | 0^b^ |  |  | 0 |  |  |  |  |
| ^a^ The reference category is: ‘No Change’.  ^b^ This parameter is set to zero because it is redundant | | | | | | | | | | | | | | | | | |
| * N= 213. The table shows the analysis of 213 respondents out of 250. 37 women had the answer to this question about the change of marital relationship as inapplicable since they were not married when diagnosed with BC. | | | | | | | | | | | | | | | | | |

**Supplementary Table 2.** Multinomial logistic regression analysis of survivors’ changes in marital life by socio-demographic, illness-related and support-related variables (N=213*)
